# Adapting and testing measures of organizational context in primary care clinics in KwaZulu-Natal, South Africa

**DOI:** 10.1186/s12913-024-11184-9

**Published:** 2024-06-18

**Authors:** Hannah H. Leslie, Sheri A. Lippman, Alastair van Heerden, Mbali Nokulunga Manaka, Phillip Joseph, Bryan J. Weiner, Wayne T. Steward

**Affiliations:** 1 Division of Prevention Science, Department of Medicine, University of California, San Francisco, San Francisco, USA; 2 MRC/Wits Rural Public Health and Health Transitions Research Unit (Agincourt), School of Public Health, Faculty of Health Sciences, University of the Witwatersrand, Johannesburg, South Africa; 3 Division of Human and Social Capabilities, Human Sciences Research Council, Durban, South Africa; 4 Department of Paediatrics, School of Clinical Medicine, Faculty of Health Sciences, SAMRC/WITS Developmental Pathways for Health Research Unit, University of the Witwatersrand, Johannesburg, South Africa; 5 Departments of Global Health and Health Systems and Population Health, University of Washington, Seattle, USA

**Keywords:** Organizational context, South Africa, Primary care, Leadership, Stress, Cohesion, Critical consciousness, Instrument development, Reliability, Validity

## Abstract

**Background:**

Implementation science frameworks situate intervention implementation and sustainment within the context of the implementing organization and system. Aspects of organizational context such as leadership have been defined and measured largely within US health care settings characterized by decentralization and individual autonomy. The relevance of these constructs in other settings may be limited by differences like collectivist orientation, resource constraints, and hierarchical power structures. We aimed to adapt measures of organizational context in South African primary care clinics.

**Methods:**

We convened a panel of South African experts in social science and HIV care delivery and presented implementation domains informed by existing frameworks and prior work in South Africa. Based on panel input, we selected contextual domains and adapted candidate items. We conducted cognitive interviews with 25 providers in KwaZulu-Natal Province to refine measures. We then conducted a cross-sectional survey of 16 clinics with 5–20 providers per clinic (*N* = 186). We assessed reliability using Cronbach’s alpha and calculated interrater agreement (a_wg_) and intraclass correlation coefficient (ICC) at the clinic level. Within clinics with moderate agreement, we calculated correlation of clinic-level measures with each other and with hypothesized predictors – staff continuity and infrastructure – and a clinical outcome, patient retention on antiretroviral therapy.

**Results:**

Panelists emphasized contextual factors; we therefore focused on elements of clinic leadership, stress, cohesion, and collective problem solving (critical consciousness). Cognitive interviews confirmed salience of the domains and improved item clarity. After excluding items related to leaders’ coordination abilities due to missingness and low agreement, all other scales demonstrated individual-level reliability and at least moderate interrater agreement in most facilities. ICC was low for most leadership measures and moderate for others. Measures tended to correlate within facility, and higher stress was significantly correlated with lower staff continuity. Organizational context was generally more positively rated in facilities that showed consistent agreement.

**Conclusions:**

As theorized, organizational context is important in understanding program implementation within the South African health system. Most adapted measures show good reliability at individual and clinic levels. Additional revision of existing frameworks to suit this context and further testing in high and low performing clinics is warranted.

**Supplementary Information:**

The online version contains supplementary material available at 10.1186/s12913-024-11184-9.

## Background

Despite the large investment in research to identify clinical and behavioral interventions to improve HIV prevention and care, many efficacious programs never get incorporated into policy or scaled into clinical settings; others fail when put into practice [1, 2]. In contexts such as South Africa, with 7.7 million people living with HIV (PLHIV), 4.8 million on antiretroviral therapy (ART) [3], and an aging population of PLHIV who have increasingly complex care needs [4, 5], scaling interventions that ensure effective, evidence-based care is a priority [6]. To this end, the field of implementation science has begun to shed light on why some efficacious interventions have not translated into programmatic successes, noting factors that must be addressed within the clinical environment to improve implementation and sustainment [1, 7, 8].

Implementation science frameworks situate interventions within the organizational context of a health care setting. The Exploration, Preparation, Implementation, Sustainment (EPIS) conceptual framework includes absorptive capacity, culture, climate, and leadership as elements of the context that shape exploration of interventions [9, 10], while the Consolidated Framework for Implementation Research (CFIR) identifies domains such as culture, implementation climate, and readiness for implementation as key factors at the organizational or team level [11, 12]. Recent updates to CFIR have focused on clarifying these domains as antecedents on the pathway to implementation outcomes [12]. The theory of Organizational Readiness for Change similarly identifies contextual factors such as the culture and climate of the organization that help to shape readiness for a specific change, which in turn affects implementation effectiveness [13]. Researchers have drawn on these definitions in efforts to better measure organizational characteristics: a 2017 systematic review found 76 articles attempting to measure organizational context, a majority of which were based in the United States; the authors recommended greater efforts to use mixed-methods research to develop and test measures in a range of settings [14].

Measures developed within the US health care system reflect the decentralized nature of the system, the national culture of individualism, and high levels of clinical autonomy that distinguish the US health care system from that of many other nations with more hierarchical, top-down power structures. The lack of validated measures of organizational context in centralized health systems, particularly in low-resource countries where primary care clinics are overextended, contributes to a clear gap in understanding which contextual factors impact successful program implementation and how these factors can be addressed [15, 16]*.* Research in South Africa from our team and others has found that program implementation can be heavily influenced by clinic leadership, particularly leaders’ problem-solving skills, in addition to provider teamwork and clinic environment such as material and human resources [17–20]. Qualitative assessment across multiple levels of the health system in KwaZulu-Natal Province identified perceived benefits of a particular program as well as broader resource availability and clear communication as factors shaping integration of HIV programming into general care [21]. Recent research on implementation of maternal health quality improvement underscored the importance of leadership, teamwork, and provider motivation in maintaining consistent implementation of interventions, particularly in the face of external factors such as the COVID-19 pandemic, budget cuts, and labor actions [20].

In this study, we aimed to adapt implementation science frameworks to the context of primary care in South Africa, to develop and test measures of organizational context based on the adapted framework, and to assess if the resulting measures demonstrated associations with hypothesized determinants and outcomes of organizational context.

## Methods

We report this study, which included formative qualitative work and a cross-sectional survey, based on recommendations for scale development and testing studies [22] and following STROBE guidelines for observational research (Additional file 1).

### Study setting

This study took place in uMgungundlovu District in KwaZulu-Natal Province, South Africa. The district includes the capital city of Pietermaritzburg but is otherwise largely rural. Adult HIV prevalence is estimated at 30%, and the 57 Department of Health (DOH) facilities provide ART for approximately 140,000 individuals [23, 24]. The U.S. President’s Emergency Plan for AIDS Relief (PEPFAR) supports the national HIV response in this district by funding implementing partner organizations that second staff to DOH facilities in support of HIV care and that provide specific services like clinical mentoring or client tracing following disengagement from care.

### Domain and item generation

**Fig. 1:**
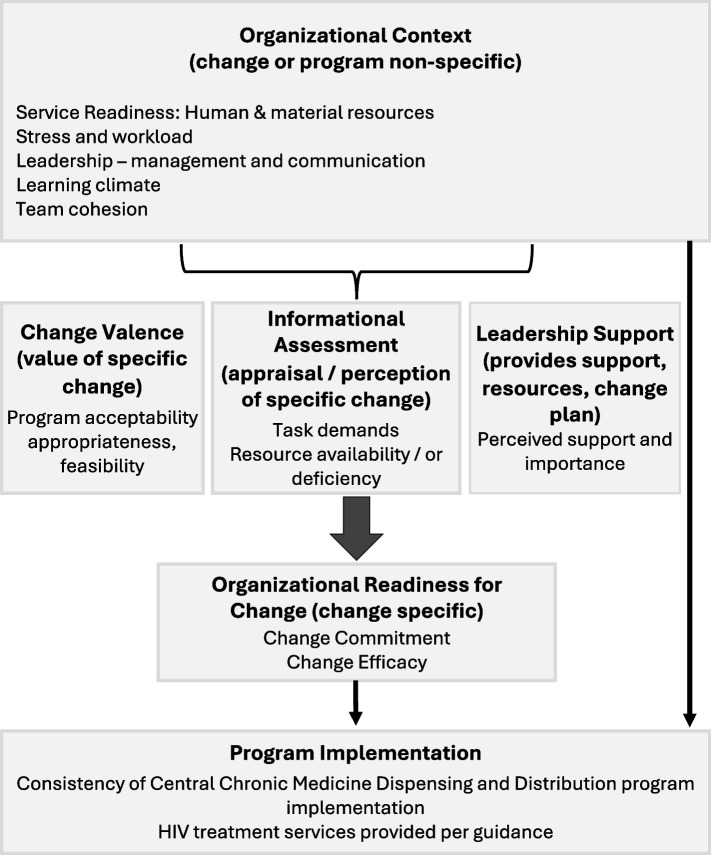
Original conceptual model of factors shaping program implementation

We followed an iterative approach to define priority domains and identify potential items. We first synthesized implementation science literature and existing research in South Africa into a conceptual model delineating organizational context as an overall determinant of program-specific domains and ultimately organizational readiness for change (Fig. 1). We defined key elements of organizational context as service readiness (the resources available for service provision), stress and workload, leadership (including leadership practice, communication, and direction on roles and responsibilities), learning climate as a space for shared trial and evaluation, and team cohesion (trust and shared values). Primary care clinics in this area typically operate with an Operational Manager (OM) who is also a professional nurse, a deputy manager, and a small number of nurses who rotate through clinical services; we initially conceptualized the full clinic as the organizational unit, the OM and deputy as leaders, and the nursing and support staff as the relevant team. We used literature searches, investigator knowledge and networks, and the Community Advisory Board of the Human Sciences Research Council to identify participants for an Expert Panel composed of health care providers, program managers, social scientists, and DOH representatives all familiar with the provision of HIV care in the province. We convened the panel in a hybrid format and presented the initial conceptual framework for their review. The Expert Panel affirmed the primacy of organizational context in shaping program implementation in this setting, and noted major aspects to consider: that program implementation and sustainment is driven by top-down directives, that the context of overburdened facilities and resource constraints shapes program uptake, that leadership communication and management of complex relationships within and beyond facilities is critical, and that providers face high demands and shifting roles that can make teamwork particularly important. Panelists noted that the concept of a learning climate informed by a quality improvement feedback loop was not present within primary facilities; they highlighted leadership’s role in monitoring performance as more salient, complemented by providers’ active interest in solving implementation problems. Panelists concurred with conceptualizing clinics as an organizational unit with distinct leaders and a single team of providers. Following the Panel discussion, we returned to the literature to clarify candidate constructs and identify potential items. We also mapped service readiness to specific human and material resources that inform organizational context for the full clinic.

We identified 7 latent constructs for measurement, including 4 related to leadership; Table 1 defines each construct and the accompanying measure. Scales on leadership engagement, feedback and monitoring, and stress addressed our revised conceptual framework, and each was based on an existing measure validated in other contexts [25, 26]. To measure teamwork, we adapted a measure of cohesion we had previously validated in community settings in South Africa.[27] In place of directly assessing “learning climate”, we adapted a measure on critical consciousness, which we had originally developed to capture community empowerment and learning culture [27, 28], to address problem solving within the facility as described by the Expert Panel. Two scales were not direct adaptations. Based on other research in South African clinics [17] and Expert Panel input on the importance of leaders in optimizing implementation in light of resource constraints, we drew from and expanded on existing items on change efficacy [29, 30] to create a leadership-focused scale on resource mobilization and problem solving. To capture coordination in this setting, we developed new items to capture the specifics of coordinating facility staff, implementing partners, and community leaders. The Expert Panel reviewed and revised items for clarity; we reconvened a subset of the panel to finalize items collectively. Items were translated into isiZulu and back translated to English by co-author MM.

**Table 1:** Organizational context measures

Construct	Definition	Sample item	Primary source
Leadership measures			
Leadership engagement	Commitment, involvement, and accountability of leaders and managers	Clinic leadership creates an environment where work can be accomplished	CFIR inner setting, leadership engagement [25]
Feedback and monitoring	Monitoring performance, making use of data to inform program implementation	Clinic leaders regularly give staff constructive feedback, with steps on how to improve	Organizational readiness to change assessment (ORCA), measurement (feedback) [26]
Resource mobilization and problem solving	Capacity to optimize resources to address problems in the face of resource constraints	Clinic leaders make the most of the staff available to implement clinic programs	Informed by: change efficacy adapted for use in Tanzanian hospital [30]
Coordination	Effective coordination of multiple stakeholders, within and beyond the clinic	Clinic leaders work effectively with implementing partners, for example HST, ANOVA, Right to Care, TB-HIV Care, or Broad Reach	Newly developed based on Expert Panel input
Non-leadership measures			
Stress	Perceived strain, stress, and role overload for individual staff	I am under too many pressures to do my job well	CFIR inner setting stress domain [25]
Cohesion	Shared trust, connectedness experienced by members of a group	Mutual trust among people who work in this clinic is strong	Social cohesion within communities in South Africa[27]
Critical consciousness	Degree to which providers and staff in a clinic actively work together to problem solve	People in the clinic not only talk about problems but also try to solve them	Critical consciousness within communities in South Africa[27]

We conducted cognitive interviews with 25 participants in 2 stages from June to September 2021 for content validation of the proposed items, allowing time to revise between stages. We sampled 19 providers (primarily nurses) and 6 organizational managers (OMs, clinic leaders) from 5 facilities and tested 3 to 4 scales per interview. We selected nurses and OMs for cognitive interviews based on their central responsibility for delivering care and hence capacity to answer all proposed items, including items on clinical care delivery not included in this analysis. We used the think-aloud method and probed respondents on clarity of the item and response choices, thought processes leading to their response, and ease of answering. We asked respondents to identify overlapping or redundant items. Interviews were conducted in English or isiZulu. We recorded interviews and translated and transcribed for analysis. We conducted rapid analysis to assess key terms such as “clinic leaders” and “clinic staff,” and we iteratively revised items and scales for clarity and efficiency. For instance, we revised an item on whether “leaders make use of all available staff to implement clinic programs” to whether “leaders make the most of the staff available” based on responses that not all staff are needed or appropriate for a given program. Interviewees provided consistent responses in conceptualizing their clinics as a single unit, identifying the OM and deputy as the relevant leaders, and defining nursing and support staff personnel as a team. The final instrument included 4 or 5 items per scale with 4 response options for each statement (See Additional file 2, Table S1 for all items). Greater agreement indicated respondents perceived more of that construct within the clinic; all constructs except for stress were positive aspects of organizational culture and most items on these constructs had positive stems; items with negative stems were maintained as written for inter-item assessment and reverse-scored for subsequent analyses.

### Data collection

To test the proposed measures, we calculated a minimum sample size of 12 providers each within 14 facilities (168 respondents) to provide > 80% power to detect a correlation of at least 0.57 with alpha of 0.05, one-sided. To ensure this sample size while including facilities with fewer than 12 providers total, we sampled 16 facilities using random selection among facilities with at least 100 patients on ART based on provincial TIER.net data, stratified by ART patient population size (< 2000, > 2000) to account for possible differences in smaller clinics and larger clinics with potentially more complex structures. Facilities participating in cognitive interviews were ineligible. Within facility, we selected all OMs and used stratified random sampling to select up to 8 higher-level nurses (Professional Nurses, Certified Nursing Professionals, Registered Nurses) and up to 8 auxiliary nurses and other patient-facing providers engaged in HIV care (Enrolled Nurses or Enrolled Nursing Assistants, Nutritionists, Pharmacists, Nutrition or Pharmacy Assistants, Lay Counselors), as the Expert Panel and cognitive interviews confirmed that these personnel were considered part of the provider team. Selection was conducted by ordering providers at random within strata to provide replacement respondents when possible in case selected providers were not available. We administered surveys via Research Electronic Data Capture (REDCap) to capture basic demographics on providers and their roles, the organizational context measures, and additional measures on conduct of specific programs analyzed elsewhere. Data collection took place from October 2021 – March 2022. National restrictions related to the COVID-19 pandemic, including limitations on clinic scheduling and staff meetings, were shifted to alert level 1 (the lowest level) on October 1 2021 and remained at that level throughout data collection [31]; routine clinical practices continued to be affected during the study period by considerations such as diverting staff for vaccination campaigns.

We conducted a concurrent facility audit of human and material resources using direct observation and a survey with the OM or a proxy if there was no OM available. From the audit, we calculated a summary score based on the presence of key infrastructure; indicators were adapted from the World Health Organization Service Availability and Readiness Assessment and are listed in Additional file 2, Table S2 [32]. We calculated staff continuity based on the number of clinical staff and number of clinical positions with turnover in the past year. We also extracted routine program data on HIV patient outcomes from the district and national reporting systems. We calculated aggregate retention on ART per facility as the number of patients remaining on ART as of March 2022 out of the number reported on ART in March 2021 or newly starting ART between March 2021 and February 2022.

### Data analysis

Analysis proceeded in four stages. First, we conducted descriptive analysis of facilities, providers, and items. We used proportions and medians to summarize measures from the facility audit and provider surveys. We assessed incomplete responses and straightline (invariant) responses by provider and measure. Second, we conducted agreement and reliability checks at individual and facility levels. We quantified inter-item agreement with Cronbach’s alpha among complete responses. We calculated the a_wg(j)_ statistic as a measure of agreement within facility; this calculation assumes a set of parallel items answered by multiple raters and can be interpreted similarly to Cohen’s kappa. We report mean a_wg(j)_ across facilities and the number of facilities achieving at least moderate agreement (a_wg(j)_ ≥ 0.50) per measure [33]. We calculated mean respondent score per measure and used the intraclass correlation coefficient (ICC) to quantify facility-level agreement and reliability among all participants and again limited to professional nurses. Third, we conducted convergent validation using the Spearman rank correlation coefficient of all measures within facility and testing correlation with human and material resources hypothesized to shape organizational context – infrastructure and staff continuity – as well as with retention patients on ART as a clinical outcome. All validity analyses were limited to facilities demonstrating moderate agreement on the relevant measure. We assessed correlations against a pre-specified threshold of rho = 0.30 (moderate effects [34]) and reporting statistical significance. Fourth, as an exploratory analysis, we compared respondents’ average scale scores between facilities with a_wg(j)_ ≥ 0.50 (moderate agreement) on all measures versus facilities where moderate agreement was obtained on fewer measures; we used linear generalized estimating equation (GEE) models accounting for clustering within facility. All analyses were conducted in Stata version 17.

### Ethics approvals

This study was approved by the Institutional Review Board at the University of California, San Francisco (20–31802), the Research Ethics Committee at the Human Sciences Research Council (REC 1/19/08/20), and the uMgungundlovu Health District; all methods were carried out in accordance with relevant guidelines and regulations. All facilities provided consent for inclusion and each participant provided written informed consent to participate.

## Results

A representative from each of the 16 facilities consented to participation and assisted in completion of the facility audit; data from all facilities were extracted in full from district reporting. The median facility had 14 full-time clinical staff; only 3 of 16 facilities had all positions filled with permanent personnel (no vacancies, no interim posts) at the time of assessment (Table 2A). Most facilities demonstrated some gaps in core infrastructure: median score was 61%. Routine district data suggested retention on ART was high in all facilities, with a median of 95% of patients retained between March 2021 and March 2022.

**Table 2:** Characteristics of sampled facilities and providers

**A: Facilities (*N* = 16)**	Median (IQR) or N (%)
*Setting*	
Urban	6 (37.5%)
Peri-urban	2 (12.5%)
Rural	8 (50.0%)
*Road surface in 5km nearest facility*	
Gravel or stone	5 (31.2%)
Paved/tarred	11 (68.8%)
*Clinic schedule*	
Weekdays only	10 (62.5%)
Including weekend and/or overnight services	6 (37.5%)
*Human resources*	
Allocated full-time clinical staff positions	14.0 (8.0, 26.0)
% of clinical positions currently vacant	14% (0%, 33%)
All clinical staff positions filled with permanent personnel	3 (18.8%)
Continuity as % of FT clinical staff^a^	89% (73%, 93%)
*Material resources*	
Infrastructure score	61% (56%, 78%)
*Patient retention*	
Patients remaining on ART (March 2021-March 2022)^b^	95% (92%, 98%)
**B: Providers (*N* = 185)**	
*Participant Gender*	
Male	23 (12.5%)
Female	161 (87.5%)
*Professional training or provider cadre*	
Operations Manager	14 (7.6%)
Professional nurse	67 (36.2%)
Other Patient-facing^c^	104 (56.2%)
*Experience*	
Years in profession	8.0 (3.0, 10.0)
Years at facility	6.0 (1.0, 9.0)

^a^Calculated as (N clinical staff – N clinical positions turned over in past year)/ N clinical staff

^b^Based on aggregate data from the District Health Information System (DHIS)

^c^Includes: enrolled nurse and enrolled nurse assistant, lay counselor, pharmacist, pharmacy assistant, nutrition assistant

Of 194 providers approached, 186 consented to participate (95.9%); those declining cited insufficient time. One respondent who worked as a data capturer rather than a patient-facing role was excluded from analysis. Consistent with health care providers in South Africa generally, most respondents were female (87.5%, Table 2B). Due to extensive turnover in leadership of some clinics, surveys were completed by OMs at 14 of 16 facilities, including one interim OM. Just over one third of respondents were non-OM nurses. Respondents reported a median of 8 years of professional experience and 6 years at their current facility.

At the individual level, respondents tended to agree with most items: average scores for the proposed measures clustered near 3 = “Agree” out of the possible range 1 – 4 (Table 3). Straightline responses were common, ranging from 34% of respondents on the measure of stress to 54% for critical consciousness; nearly all such responses were all “Agree” except for the measure on stress, where straightline responses were split between “Agree” and “Disagree” (data not shown). Leadership coordination had the highest missingness, with 59 participants responding “Don’t know” or skipping at least one item, primarily two items related to the external clinic committee (whether the committee met regularly and if leaders acted on its input). Cronbach’s alpha indicated moderate to strong inter-item agreement for all measures except coordination. We excluded coordination from subsequent analysis given the high degree of missingness and inadequate inter-item agreement.

**Table 3:** Scale performance and measurement properties

A: Individuals						
	Items	Complete N	Mean ± SD	Score range (out of 1 – 4)	Straightline responses	Alpha
Leadership engagement	5	181	2.97 ± 0.46	1.4 – 4.0	48%	0.83
Feedback and monitoring	4	182	3.08 ± 0.44	2.0 – 4.0	48%	0.77
Resource mobilization	5	180	2.81 ± 0.42	1.4 – 4.0	36%	0.73
Coordination	4	126	3.01 ± 0.37	2.0 – 4.0	49%	0.65
Stress	5	183	2.73 ± 0.59	1.4 – 4.0	34%	0.86
Cohesion	5	183	2.88 ± 0.49	1.2 – 4.0	36%	0.83
Critical consciousness	5	180	2.95 ± 0.40	1.8 – 4.0	54%	0.84
B: Facility level^a^						
	ICC (*N* = 185)	ICC, nurses (*N* = 81)		Facility a_wg(j)_ mean (*N* = 16)	Facilities w/ moderate agreement, a_wg(j)_ ≥ 0.50 (*N* = 16)	
Leadership engagement	0.00	0.02		0.68	14	
Feedback and monitoring	0.06	0.19		0.68	15	
Resource mobilization	0.03	0.15		0.69	13	
Stress	0.23	0.32		0.57	12	
Cohesion	0.14	0.05		0.67	13	
Critical consciousness	0.22	0.32		0.77	16	

^a^Coordination not analyzed at facility level due to missingness and inadequate inter-item agreement

Facilities accounted for up to 22 to 23% (for critical consciousness and stress, respectively) of total variance in mean scores. ICC exceeded a minimum threshold of 0.05 for feedback and monitoring, stress, cohesion, and critical consciousness (Table 3B); near zero ICC for leadership engagement and resource mobilization suggested these measures could not reliably distinguish between facilities. ICC was higher when limited to professional nurses for most measures, suggesting that for measures other than leadership engagement, professional nurses responded more consistently within facilities than other providers. Distribution of facility means underscores the homogeneity of scales like leadership engagement across all facilities (Additional file 2, Figure S1).

Item responses demonstrated moderate to strong agreement within facilities, with a_wg(j)_ ranging from 0.57 for stress (12 of 16 facilities with at least moderate agreement) to 0.78 for critical consciousness (all facilities with at least moderate agreement). Facilities with a_wg(j)_ < 0.50 demonstrated inconsistent agreement across responses to consider a summary statistic representative of the facility as a whole.

Limiting analysis to facilities with at least moderate agreement on a given measure, we found that the 6 measures of organizational context showed substantial correlation within facility: absolute correlation exceeded the predetermined threshold of 0.30 in all cases and achieved statistical significance at *p* < 0.05 for multiple assessments despite the small number of facilities (Table 4A). When compared with predicted inputs and outcomes of facility climate (Table 4B), correlation with staff continuity was moderate (*rho* > 0.30) for feedback and monitoring, resource mobilization, and stress, with only stress showing a statistically significant correlation (-0.68). Higher scores on feedback and monitoring were correlated with lower facility infrastructure (*rho* = -0.53), contrary to expectation. Cohesion was correlated with higher retention on ART (*rho* = 0.49, *p* = 0.09).

**Table 4:** Correlation of facility-level measures within facilities with moderate agreement (a_wg(j)_ ≥ 0.50)

	Leadership engagement		Feedback and monitoring		Resource mobilization		Stress	Cohesion
Feedback and monitoring	**0.59** ***N* = 13** ***p* = 0.034**							
Resource mobilization	**0.81** ***N* = 11** ***p* = 0.003**		**0.42** ***N* = 13** ***p* = 0.149**					
Stress	**-0.71** ***N* = 11** ***p* = 0.014**		**-0.66** ***N* = 11** ***p* = 0.028**		**-0.55** ***N* = 11** ***p* = 0.082**			
Cohesion	**0.74** ***N* = 11** ***p* = 0.009**		**0.34** ***N* = 12** ***p* = 0.285**		**0.80** ***N* = 11** ***p* = 0.003**		**-0.59** ***N* = 11** ***p* = 0.056**	
Critical consciousness	**0.70** ***N* = 14** ***p* = 0.005**		**0.52** ***N* = 15** ***p* = 0.0.04**		**0.92** ***N* = 13** ***P* < 0.001**		**-0.46** ***N* = 12** ***p* = 0.137**	**0.76** ***N* = 13** ***p* = 0.003**
B: Facility inputs and patient outcomes								
	**Inputs**				**Outcomes**			
	**Staff continuity**		**Infrastructure**		ART retention			
	Rho (*p* value)		Rho (*p* value)		Rho (*p* value)			
Leadership engagement (*N* = 14)	0.25 (0.398)		0.08 (0.797)		0.18 (0.541)			
Feedback and monitoring (*N* = 15)	**0.38 (0.159)**		**-0.53 (0.042)**		0.05 (0.869)			
Resource mobilization (*N* = 13)	**0.46 (0.117)**		0.05 (0.865)		-0.22 (0.471)			
Stress (*N* = 12)	**-0.68 (0.016)**		0.27 (0.391)		-0.28 (0.372)			
Cohesion (*N* = 13)	0.07 (0.830)		0.07 (0.816)		**0.49 (0.090)**			
Critical consciousness (*N* = 16)	0.24 (0.377)		-0.16 (0.547)		0.11 (0.672)			

*Bolding signifies abs(rho)* > *0.30*

In our exploratory analysis to understand potential differences in context in facilities where staff were largely in agreement on their scoring, we found that respondents in the 9 facilities with moderate agreement on all scales reported more positive organizational context than the other 7 facilities, with statistically significant differences in leadership engagement, resource mobilization, and stress (Table 5). The largest difference was in reported stress: average scores on the stress scale were 0.41 points lower (less stress) in facilities with agreement on all scales.

**Table 5:** Participant responses within facilities with high overall agreement

	Average scale response		
	Facilities with fewer scales with moderate agreement (*N* = 7)	Facilities with agreement on all scales (*N* = 9)	Difference β (95% CI)
Leadership engagement	2.89	3.02	**0.13 (0.03, 0.23)**
Feedback and monitoring	3.05	3.10	0.05 (-0.10, 0.20)
Resource mobilization	2.75	2.86	**0.11 (0.02, 0.21)**
Stress	2.96	2.55	**-0.41 (-0.65, -0.17)**
Cohesion	2.84	2.92	0.08 (-0.09, 0.26)
Critical consciousness	2.90	3.00	0.10 (-0.07, 0.27)

## Discussion

In this study, we developed and adapted measures for 7 domains of organizational context based on implementation science frameworks and expertise within primary care clinics in South Africa. The measures demonstrated reasonable individual-level consistency with our study population, except for the coordination scale created de novo; the remaining measures showed moderate to strong agreement and low to moderate reliability within facility. Variance between facilities was modest, possibly reflecting the shared context of a rural setting in a single district. Measures generally correlated with each other at the facility level, though we found limited evidence of relationships between the facility scores and hypothesized predictors and outcomes in validation analyses. Facilities with stronger agreement among respondents also tended to have a more positive context.

The Expert Panel concurred with existing literature and implementation science frameworks that facility leadership was critical to program implementation and sustainment. In this setting of relatively small clinics and distribution of responsibilities across staff, they prioritized overall leadership above leadership specific to implementation of one program, which has been more commonly measured in US-based implementation science research [35]. We adapted or developed measures for four aspects of overall leadership hypothesized to improve program implementation: engagement, feedback and monitoring, resource mobilization, and coordination. The newly created items on coordination with external partners and clinic committees proved difficult for some respondents to answer and showed limited agreement even within complete responses. Further efforts to capture this important construct, potentially as an index rather than a scale, are warranted. The other leadership measures demonstrated good item agreement and moderate agreement within facilities in our sample; as measures developed for use at an organizational level, adequate agreement across raters is critical. The finding that ICCs for leadership measures were generally higher among professional nurses, typically the most trained professional cadre in primary care facilities, indicates that these providers were relatively more consistent within facility compared to across facilities than all respondents, potentially due to greater exposure to clinic leaders or to differing interpretations of who qualifies as a ‘leader’ between professional nurses and other personnel. Our cognitive interviews demonstrated consistent understanding of ‘leader’ among the professional nurses and OMs we interviewed; including additional cadres could be useful to extend this evidence. The findings to date support use of these leadership measures within higher cadres such as professional nurses in similar settings, particularly for clinical interventions.

Beyond leadership, we tested measures of stress, cohesion, and critical consciousness hypothesized to shape uptake of new programs. These scales similarly demonstrated good item agreement and moderate inter-rater agreement within facilities; ICCs (0.23; 0.14; and 0.22, respectively) well exceeded the minimum threshold of 0.05 among all participants, suggesting greater consistency in respondents across cadres within facility than for leadership measures.

The six scales demonstrating sound measurement properties also tended to correlate together within facilities, potentially reflecting less random variation in these facilities and/or that respondents provided similar responses across scales and between raters within these facilities. Three scales demonstrated some correlation with inputs and outputs in accordance with predictions: resource mobilization, stress, and cohesion. Better resource mobilization was correlated with higher staff continuity. The scale for stress, the only construct indicating a negative climate and where agreement indicated worse performance, had less homogeneity than other scales, surfacing potential to further refine the measure to better distinguish between clinic contexts. Higher stress also showed a correlation with lower staff continuity. The scale for cohesion – teamwork within providers – demonstrated moderate heterogeneity between individuals and between facilities, and was correlated with retention on ART. Given the importance of provider burnout before and especially during the COVID-19 pandemic [36–38], better assessment of stress and cohesion can help to identify best performing clinics and to target facilities most in need of management or individual interventions on fostering teamwork and coping with stress.

The remaining scales—on leadership engagement, feedback and monitoring, and critical consciousness—demonstrated two drawbacks. The first was high levels of straightline responses, with approximately half of respondents indicating the same answer—typically “Agree”—to all items. These response patterns could be explained in several ways: 1) truly uniform conditions across and within these facilities within a single district, 2) insufficient distinction between items to capture indications of very low or very high levels of each construct, 3) social desirability within a hierarchical work setting, 4) lack of strong opinion, particularly given the strain providers face to deliver care amidst constrained resources, and/or 5) respondent inattention or fatigue. In the absence of a neutral response option (which we did not provide to avoid respondents defaulting to the median option), one or more of these explanations could have resulted in repeatedly agreeing. This degree of invariance can inflate apparent agreement within individuals and facilities, but it undermines the utility of the measures in distinguishing between facilities, should such distinctions exist.

The second drawback was inconsistent evidence of correlation with hypothesized predictors (staff continuity and infrastructure) and with patient outcomes (retention on ART) in the validation analysis. Careful consideration is required to understand these findings. It is possible that these initial efforts to adapt the constructs of organizational context did not fully capture the dynamics that most strongly shape performance in these facilities. This may be particularly salient given the time of the assessment following the upheaval of the COVID-19 pandemic, which shaped staff continuity, organizational context, and ART retention. An additional limitation is the relative insensitivity of the outcome measure: ART programs are longstanding, and our measure of patient retention based on (imperfect) aggregate data demonstrated little variability across the sampled facilities. Organizational context at the time of assessment may have had little influence on patient retention even had it been measured perfectly. Measures reflecting implementation or sustainment of more recently introduced programs would provide an indicator more sensitive to variation in organizational context.

Our study has multiple strengths, including use of implementation science frameworks and organizational readiness theory to propose measures of organizational context in primary care facilities in South Africa. This work expands on the qualitative work attesting to the importance of organizational context in implementing and maintaining interventions in this setting [17, 20, 21, 39]. We relied on a majority South African Expert Panel to prioritize constructs and items for measurement, and we conducted detailed cognitive interviews to revise and clarify items. Limitations include difficulty in reaching providers – particularly clinic managers – amidst regular turnover and COVID-19 challenges (including the rapid changes in clinical responsibilities and locations, provider illnesses and deaths, and restrictions on routine activities) and the reliance on aggregate patient outcome data that were both imperfect and potentially insensitive to organizational context. Soliciting perspectives on leadership and organizational context in hierarchical settings is inherently fraught; it is difficult to disentangle social desirability from true agreement. Thresholds for agreement measures are imposed on a continuous metric and may not distinguish truly different performance levels [33].

## Conclusions

This study was an initial effort to adapt and test measures of organizational context to better understand program implementation in primary care within the South African health system. This work confirms the importance of organizational context from the perspective of those working within primary care clinics and supports standing calls for further efforts to develop and test theories, frameworks, and measures that capture the dynamics of health settings in resource-constrained settings. While this initial effort at adaptation of theory and measurement to the context of South African clinics produced scales with sound measurement properties and several scales – notably resource management, stress, and cohesion – with promise for differentiating facilities, further work is needed to understand the most important domains of organizational context shaping patient outcomes. From there, further efforts to refine constructs and measures are warranted, including positive deviance assessments to ensure a sample of facilities with strongly divergent performance and inclusion of implementation outcomes more closely tied to organizational context.

### Supplementary Information

Supplementary Material 1.

Supplementary Material 2.

## Data Availability

The datasets used during the current study are available from the corresponding author on reasonable request.

## Abbreviations

ART: Antiretroviral
a_wg_: Agreement within group
CFIR: Consolidated Framework for Implementation Research
COVID-19: Coronavirus disease 2019
DOH: Department of Health
EPIS: Exploration, Preparation, Implementation, Sustainment
GEE: Generalized estimating equation
HIV: Human immunodeficiency virus
ICC: Intraclass correlation coefficient
OM: Operational manager
PEPFAR: President’s Emergency Plan for AIDS Relief
REDCap: Research Electronic Data Capture
