# Supplementary material for: Adapting and testing measures of organizational context in primary care clinics in KwaZulu-Natal, South Africa
Source: BMC Health Serv Res. 2024 Jun 18;24:744. doi: 10.1186/s12913-024-11184-9 (PMC11184827; doi:10.1186/s12913-024-11184-9)

**Additional file**

**Table S1: Scale items**

Response options: Strongly agree, agree, disagree, strongly disagree

| **Leadership: engagement** |
| --- |
| Clinic leadership makes sure that we have the opportunity to discuss changes to improve care |
| Clinic leadership creates an environment where work can be accomplished |
| Clinic leadership promotes an environment that is a pleasant place to work |
| Clinic leadership provides support and guidance for people working at this clinic |
| Clinic leadership encourages changes in practice to improve patient care |
| **Leadership: feedback and monitoring** |
| Clinic leaders, such as the Operations Manager, regularly provide staff with information on clinic performance relative to targets |
| Clinic leaders regularly hold staff members accountable for achieving results |
| Clinic leaders regularly give staff constructive feedback, with steps on how to improve |
| Clinic leaders praise and regularly recognize staff for a job well done |
| **Leadership: resource mobilization and problem solving** |
| Clinic leaders make the most of our resources and supplies when we have to implement new programs. |
| Clinic leaders make the most of the staff available to implement clinic programs. |
| Clinic leaders help staff to prioritize the most important tasks to get done. |
| Clinic leaders do not provide adequate support when problems arise. |
| Clinic leaders can manage staff conflicts effectively. |
| **Leadership: coordination** |
| Clinic leaders work effectively with community [partners / stakeholders]. |
| Clinic leaders work effectively with implementing partners, for example HST, ANOVA, Right to Care, TB-HIV Care, or Broad Reach. |
| The clinic committee meets on a regular schedule. |
| Clinic leaders consistently act on feedback from the clinic committee. |
| **Stress** |
| I am under too many pressures to do my job well. |
| People who work at this clinic often show signs of stress and strain. |
| The heavy workload here reduces the quality of care for patients. |
| I am pulled in too many directions to do my job effectively. |
| Frequent change in staff responsibilities is a challenge for this clinic. |
| **Team cohesion and problem solving** |
| People who work in this clinic are usually quick to help one another when needed. |
| Mutual trust among people who work in this clinic is strong. |
| There is often tension among people who work at this clinic. |
| People who work in this clinic feel like part of a team. |
| People who work in this clinic share similar goals for the clinic. |
| **Critical consciousness** |
| People in this clinic work together to solve problems. |
| People in the clinic willingly volunteer to help solve clinic problems. |
| Staff think about *why* there are problems so that they can address them. |
| People in the clinic not only talk about problems but also try to solve them. |
| If initial efforts fail to address a problem, people in the clinic will try a different approach. |

**Table S2: indicators used from facility audit to quantify infrastructure**

| Functional phone (land line or mobile phone supported by the facility) |
| --- |
| Functioning computer |
| Internet: accessible via a facility device, paid for by the facility, with few or no interruptions during working hours, past 7 days |
| Electricity always available from main or backup source, past 7 days |
| Functional ceiling fan |
| Functional air conditioning |
| Running water available with no lapse > 2 hours, past 7 days |
| A room with auditory and visual privacy available for patient consultations |
| Toilet (latrine) on the premises in functioning condition that is accessible for general outpatient client use and has soap and running water for hand washing at time of the audit. |

**Figure S1: Average scale scores by facility**


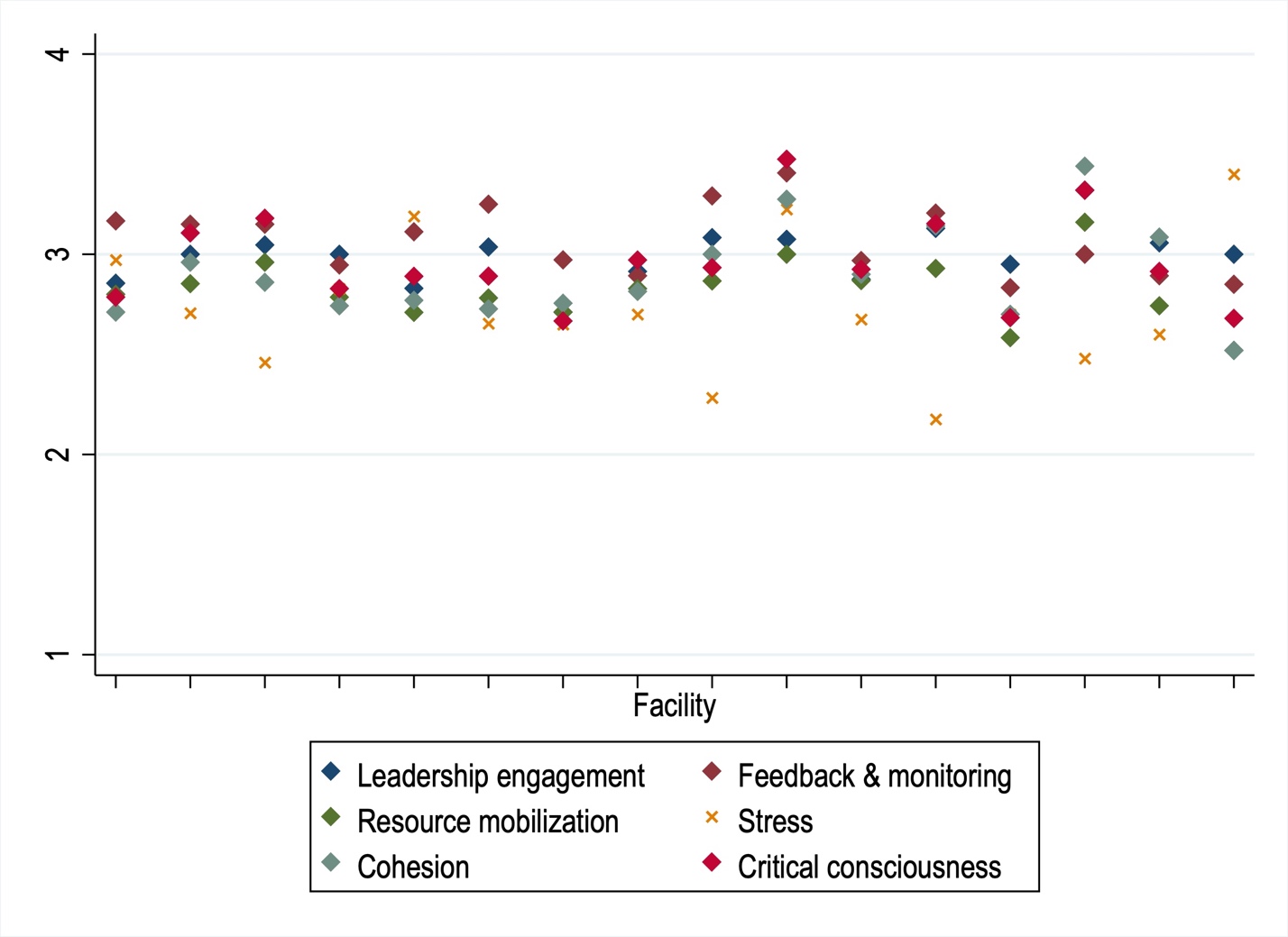

Supplement: Supplementary file 2 — Supplementary Material 2. [file 12913_2024_11184_MOESM2_ESM.docx]
